# Prospects of nanotechnology and natural products for cancer and immunotherapy

**DOI:** 10.3762/bjnano.16.116

**Published:** 2025-09-22

**Authors:** Jan Filipe Andrade Santos, Marcela Bernardes Brasileiro, Pamela Danielle Cavalcante Barreto, Ligiane Aranha Rocha, José Adão Carvalho Nascimento Júnior

**Affiliations:** 1 Department of Pharmacy, Federal University of Sergipe, São Cristóvão, Brazil; 2 Laboratório de Ensaios Farmacêuticos e Toxicidade, Federal University of Sergipe, São Cristóvão, Brazil; 3 Postgraduate Program in Health Sciences, Federal University of Sergipe, Aracaju, Sergipe, Brazil

**Keywords:** cancer, immunotherapy, nanotechnology, natural products, patent, review

## Abstract

Nanotechnology is revolutionizing pharmaceutical industry and drug development by providing significant advantages in controlling drug release, enhancing stability, and reducing adverse effects. Concurrently, natural products are being extensively researched for their anticancer and immunomodulatory properties. This patent review aims to analyze publications that integrate nanotechnology with natural products to develop cancer treatments and immunotherapies. In this context, 17 patents were identified through the free online databases of the European Patent Office (EPO) and the World Intellectual Property Organization (WIPO). The review discusses various types of nanotechnology, including nanoparticles, nanocarriers, and nanocapsules, as well as bioactive compounds primarily extracted from plants. Among the most frequently identified natural products were ursolic acid, hyaluronic acid, and catechins. These bioactive compounds have been shown to promote cell cycle arrest, reduce tumor size, and exhibit synergistic effects with other anticancer agents. Consequently, the combination of natural products with nanotechnology holds significant therapeutic potential.

## Introduction

**Figure 1:**
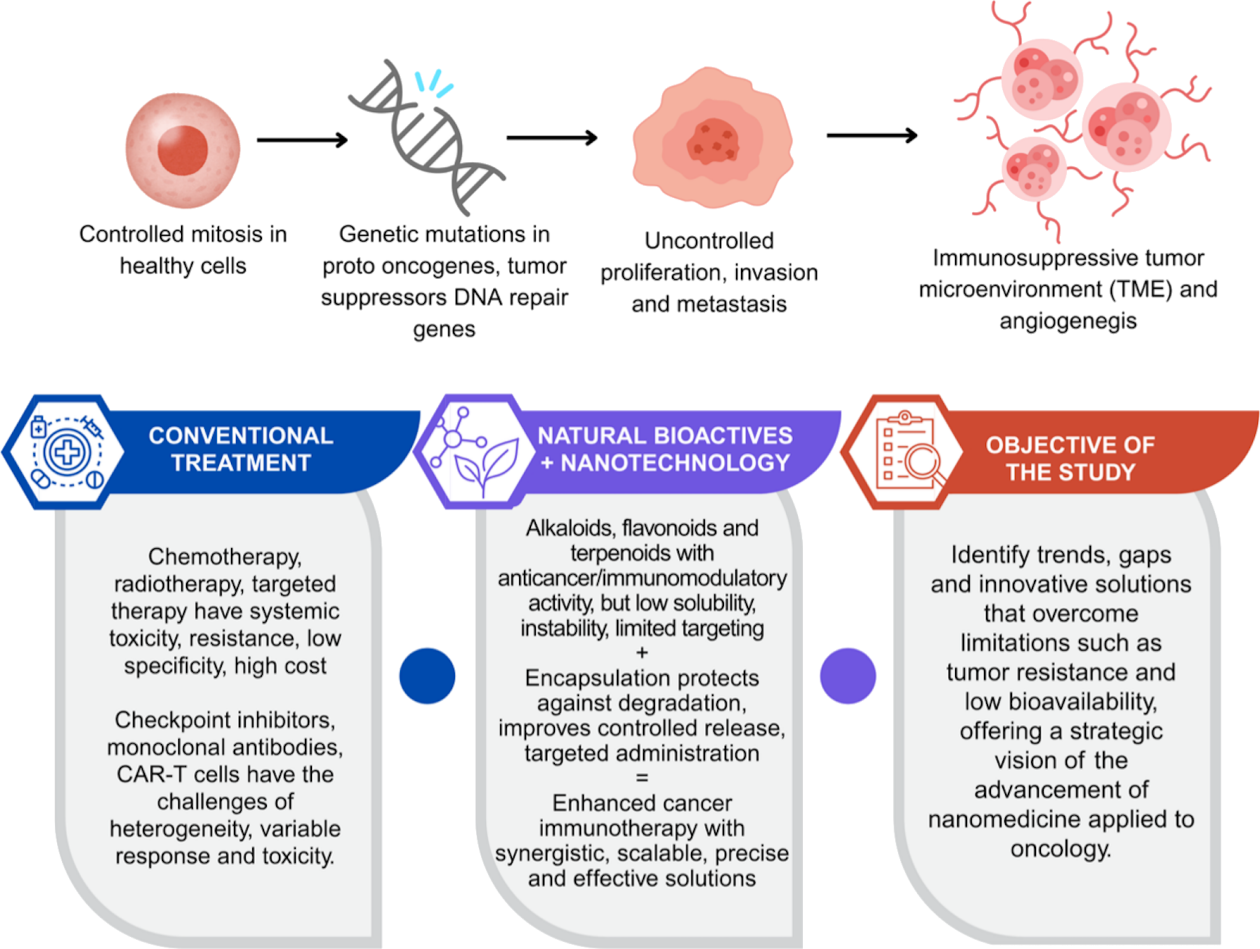
Pathophysiology of Cancer and Emerging Therapeutic Innovations. Graphical element Watercolor Illustration of a Pin: ©irasutoya via Canva.com; Graphical element Genetic Mutation Vector Icon D: ©123stock via Canva.com; Graphical element Cancer cells vector illustration: ©faridyulian via Canva.com; Graphical element cancer cell growth. cancer disease: ©surachet99 via Canva.com; Graphical element treatment outline icon: ©tulpahn via Canva.com; Graphical element Molecule Icon: ©rendicon via Canva.com; Graphical element Plant Leaves Icon: ©sirvectorr via Canva.com; Graphical element Infographic Table: ©creative-visionery via Canva.com; Graphical element Check list icon. Revision icon: ©vitaliikrasnoselskyi via Canva.com. These elements are not subject to CC BY 4.0.

Cancer is a disease characterized by the uncontrolled proliferation of abnormal cells, which have the ability to invade neighboring tissues and to metastasize to distant organs [1] (Figure 1). This pathology results from accumulated genetic alterations in proto-oncogenes, tumor suppressor genes, and DNA repair-related genes [2]. According to the International Agency for Research on Cancer (IARC), approximately 20 million new cancer cases were reported in 2022, with lung cancer being the leading cause of death, resulting in an estimated 1.8 million deaths (18.7%) during the same period [3]. The unequal distribution of incidence and mortality across different regions reflects the influence of genetic and environmental factors, while common cancer types, such as lung, breast, and prostate cancer, present significant therapeutic challenges due to their biological heterogeneity and resistance to conventional treatments [4].

The immune system is crucial for identifying and eliminating tumor cells. This highly specialized network includes cells such as T lymphocytes, dendritic cells, macrophages, and natural killer (NK) cells, as well as soluble mediators like cytokines and chemokines, which regulate inflammatory and adaptive responses [5–6]. However, cancer often employs strategies to evade the immune system, such as expressing immunosuppressive molecules and creating a hostile tumor microenvironment that suppresses antitumor activity [7]. One of the most exploited mechanisms by tumors involves immune checkpoints, such as PD-L1 (programmed death-ligand 1) and CTLA-4 (cytotoxic T-lymphocyte associated protein 4), which inhibit T-cell activation, allowing cancer cells to escape immune-mediated destruction [8].

Immunotherapy shows promise as a cancer treatment approach, encompassing strategies such as monoclonal antibodies, immune checkpoint inhibitors, antitumor vaccines, and cell-based therapies, including chimeric antigen receptor T (CAR-T) cells [9]. However, these therapies face limitations, including systemic toxicity, high cost, variable clinical responses, and tumor resistance, often associated with the genetic and phenotypic heterogeneity of tumors [10–12]. Given this scenario, bioactive compounds from natural products, such as alkaloids, flavonoids, and terpenoids, have garnered interest due to their anticancer and immunomodulatory properties [13–14]. Derivatives from plants, fungi, and microorganisms offer diverse mechanisms of action, including immune system activation and tumor growth inhibition [14]. Nevertheless, challenges like low bioavailability, chemical instability, and difficulty in targeting specific tissues hinder their effective clinical application [15].

Nanotechnology has emerged as an innovative solution to overcome the limitations of traditional therapies. Advances in cancer nanotechnology include the development of smart nanocarriers capable of responding to internal stimuli (such as pH, redox potential, and enzymes) and external stimuli (such as magnetic fields, heat, or ultrasound), enabling precise and controlled drug release [16–17]. Additionally, the use of biomimetic nanoparticles, including exosome-based delivery systems and cell membrane-coated nanoparticles, has shown promise in improving targeting efficiency and immune evasion [18–19]. Despite these advances, significant challenges remain, including nanoparticle stability in biological environments, potential immunogenicity, scalability of manufacturing processes, and the need for comprehensive long-term toxicity studies [20–21]. Overcoming biological barriers, such as penetration through the dense tumor extracellular matrix, also remains a major hurdle [19].

Nevertheless, nanotechnology opens up unprecedented opportunities in cancer immunotherapy by facilitating the co-delivery of chemotherapeutic agents, immunomodulators, and gene editing tools [22]. These multifunctional platforms can modulate the tumor microenvironment, enhance antigen presentation, reverse local immunosuppression, and improve the efficacy of cell-based therapies such as CAR-T and NK cells [23]. In this scenario, combining natural bioactive compounds with nanotechnological platforms represents a promising strategy. This approach enhances the therapeutic potential of natural molecules by improving their pharmacokinetic properties, increasing bioavailability, protecting them from degradation, and allowing for site-specific delivery [24]. Consequently, this combination addresses both the limitations of natural products and the complex challenges of cancer therapy [25].

Unlike conventional literature reviews, this study employs a technology foresight approach based on patent analysis to provide a strategic overview of emerging trends in the application of nanotechnology to natural products for cancer treatment and immunotherapy. This method allows for the identification of innovation gaps, technological barriers, and commercial opportunities, generating insights that are often overlooked in academic publications. The study systematically maps and analyzes recent patents focused on the integration of nanotechnology platforms with natural bioactive compounds in oncology and immunotherapy, highlighting technological progress and, at the same time, revealing current limitations, future directions, and perspectives for the development of effective and scalable therapeutic strategies.

## Methods

**Figure 2:**
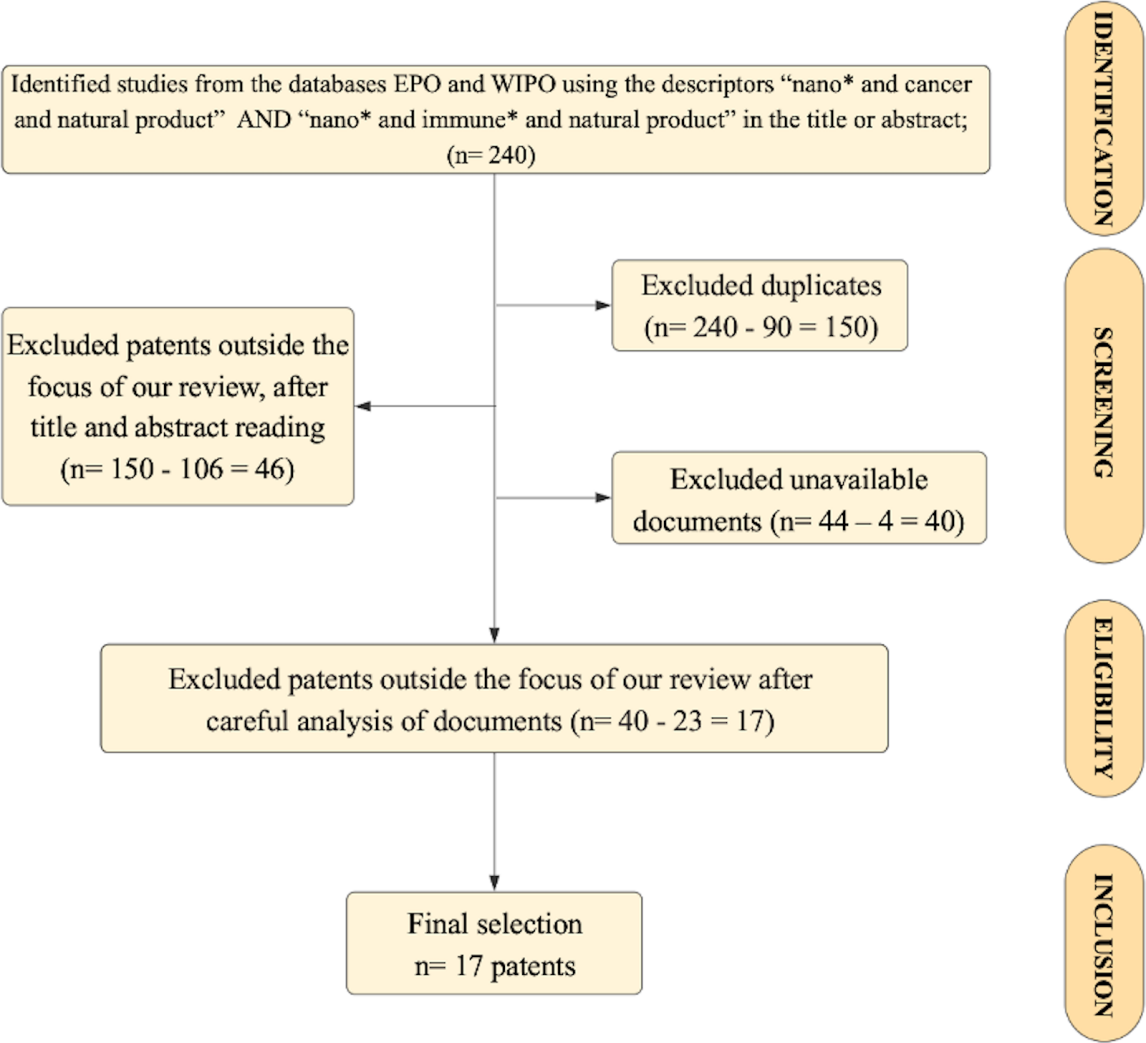
Guidelines of screening and search used in this review.

In this patent review, the free online databases of the European Patent Office (EPO) and the World Intellectual Property Organization (WIPO) were used to carry out the search using the descriptors “nano* AND cancer AND natural product” for research associated with cancer treatment, while the descriptors “nano* AND immun* AND natural product” was implemented to analyze patents related to immunotherapy. Furthermore, a time filter was applied to both surveys, with patents collected from 2016 to 2024. Given that both databases are well established and widely recognized in the field of intellectual property, and to avoid significant data duplication, we considered their inclusion sufficient for the scope of this review. In view of the subject matter addressed, the IPC classification was not used, since the search criteria implemented provided patents from different IPCs that were related to the topic addressed in the review. Also, PCT (Patent Cooperation Treaty) applications were considered, as can be seen in the inclusion of patent WO2016178224. This initial search identified 240 preliminary patents, of which 90 were excluded as duplicates. After reading the title and abstract, 106 documents were excluded due to their content being different from the focus of the review (nanotechnology formulations containing natural products for cancer treatment and immunotherapy). Subsequently, four were excluded for not having full text available. Additionally, 23 patents were eliminated for being outside the scope of the review. Finally, 17 patents were selected for critical analysis according to the objective of the study. Figure 2 shows the guidelines used for searching and screening the patents in this review, based on the PRISMA methodology.

## Results and Discussion

### Researching and screening of selected patents

**Figure 3:**
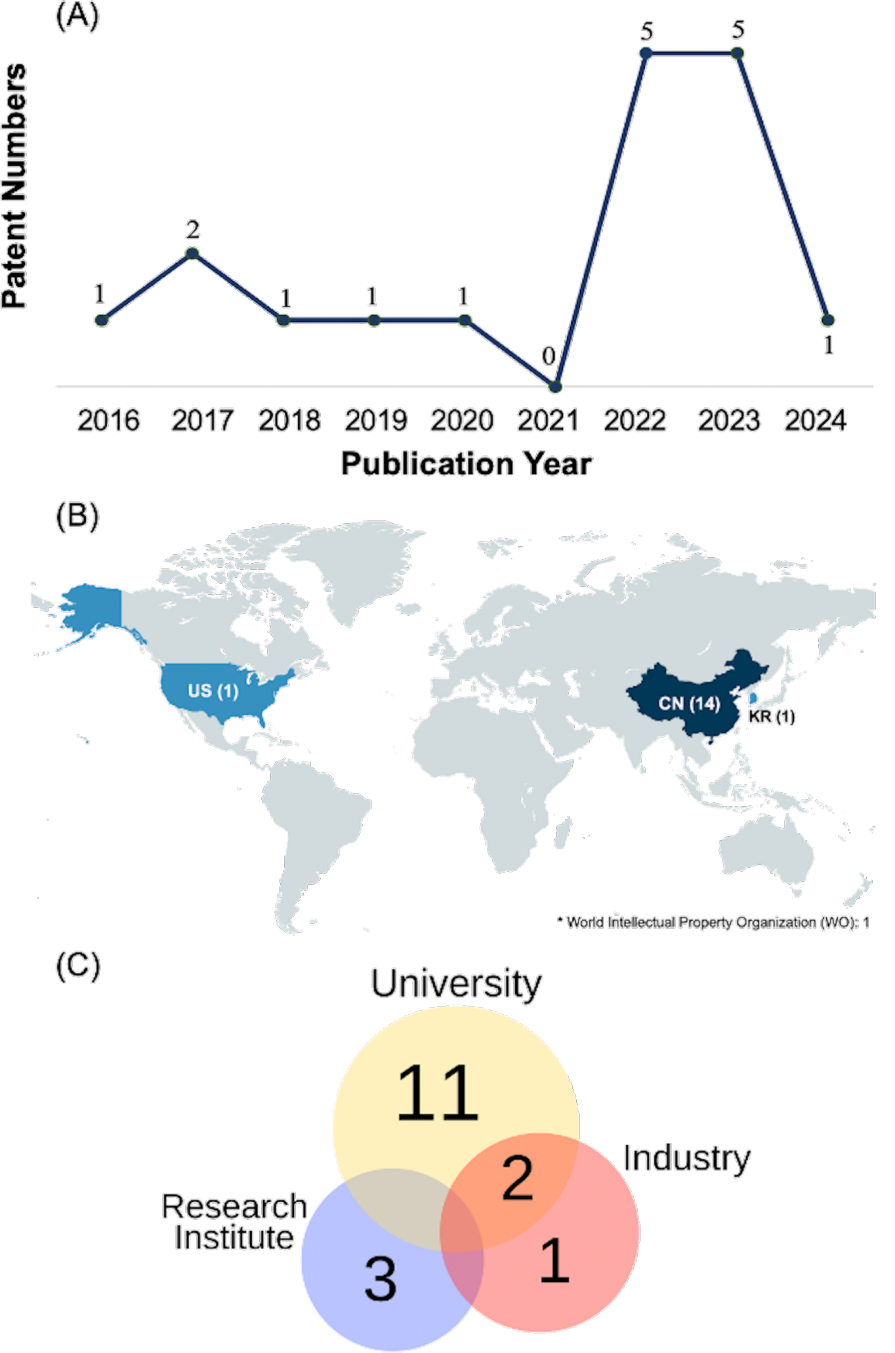
(A) Distribution of patents by publication year. (B) Distribution of patents by application country (CN: China; KR: Korea of Republic; US: United States of America; WO: World Intellectual Property Organization); (C) Distribution of patents by type of applicants. Figure 3B was created with https://www.mapchart.net/ and is distributed under the terms of the Creative Commons Attribution-ShareAlike 4.0 International License (https://creativecommons.org/licenses/by-sa/4.0/). This content is not subject to CC BY 4.0.

Based on the review criteria, the 17 selected patents were initially classified by their year of publication, covering the period from 2016 to 2024. In the first year, one document was published, a figure that remained consistent until 2021, when there was a decline, with no documents published that year. This decline can be attributed to the global impact of the COVID-19 pandemic, which began in 2020 and redirected research activities toward combating the virus. The pandemic particularly affected countries such as the United States of America and China, two major contributors to immunotherapy, related cancer research and patent production, potentially delaying research progress and patent filings. A strong recovery is evident in 2022 and 2023, with five patents published each year, suggesting a resumption of research efforts and innovation following the pandemic-induced slowdown. In 2024, the number of patents dropped again to one, although it is important to consider that data for this year may be incomplete or still in progress, especially considering the waiting time until a patent is published in the databases [26] (Figure 3A).

These results may be related to the necessity of improving treatments for health issues such as cancer. Conventional treatments like chemotherapy and radiotherapy are highly cytotoxic and nonspecific, affecting both cancerous and healthy cells. Consequently, the scientific community is seeking alternatives, one of which is the use of nanotechnology in combination with other treatments. The advent of nanotechnology has revolutionized both diagnosis and treatment, enhancing factors such as bioavailability and stability, while also reducing toxicity [27].

China (CN) leads the ranking of published inventions with 14 documents, followed by the World Intellectual Property Organization (WO), the Republic of Korea (KR), and the United States of America (USA), each with one patent (Figure 3B). The oldest selected patent was published by WO in November 2016, while the most recent was published by CN in 2024. China’s leadership in global patent activity is strongly supported by extensive public investment in research and innovation at both national and regional levels. The government offers reimbursement for patent filing and maintenance fees, along with substantial fiscal incentives, including reduced corporate income tax rates and generous super deductions for enterprises recognized as high-tech. Notably, China allocates approximately USD 1 billion annually to support scientific publications. These policy measures collectively reduce the financial barriers associated with securing patent protection, particularly in strategic sectors such as biotechnology and cancer immunotherapy, thereby reinforcing China’s prominent position in the global landscape of patent filings [28–29].

US and the Republic of Korea also have economic incentives for biotechnology research and innovation, with the US investing over 3% of its GDP in research and development and Korea allocating approximately 4.5% of its GDP to research and development [30]. Although most patents were published by the US and China, patents from other countries were also found by searching the EPO database, such as the patent KR20220169108 from South Korea, and patent WO2016178224, which is a PCT (Patent Cooperation Treaty) application.

In terms of protecting their inventions, various entities, including universities, industries, and research institutes, file for the publication of their patents. The results indicate eleven patents registered by universities such as Fuzhou University (CN), South China University of Technology (CN), Sun Yat-sen University (CN), Kyung Hee University (KR), King Abdulaziz University (US), Guizhou University of Traditional Chinese Medicine (CN), Hebei University of Technology (CN), China Three Gorges University (CN), China Pharmaceutical University (CN), South China Normal University (CN), and Sichuan University (CN). Additionally, research institutes such as Harbin Institute of Technology (CN) and The Institute of Medicinal Plant Development (CN) have published around three patents; the industry is represented by a single patent from BG Negev Technologies & Applications Ltd (WO) (Figure 3C).

The predominance of patents filed by universities in cancer research based on natural products certainly reflects the industry’s insecurity in investing in early-stage projects involving extraction, isolation, characterization, and reproducibility, as they are technically complex and costly, with little initial commercial appeal. Consequently, pharmaceutical companies tend to avoid these high-risk ventures and prefer to enter later, when the therapeutic application has already been proven, often through partnerships with universities. This trend also explains the two patents between universities and industry observed and why the industry filed only one patent [31–33].

**Figure 4:**
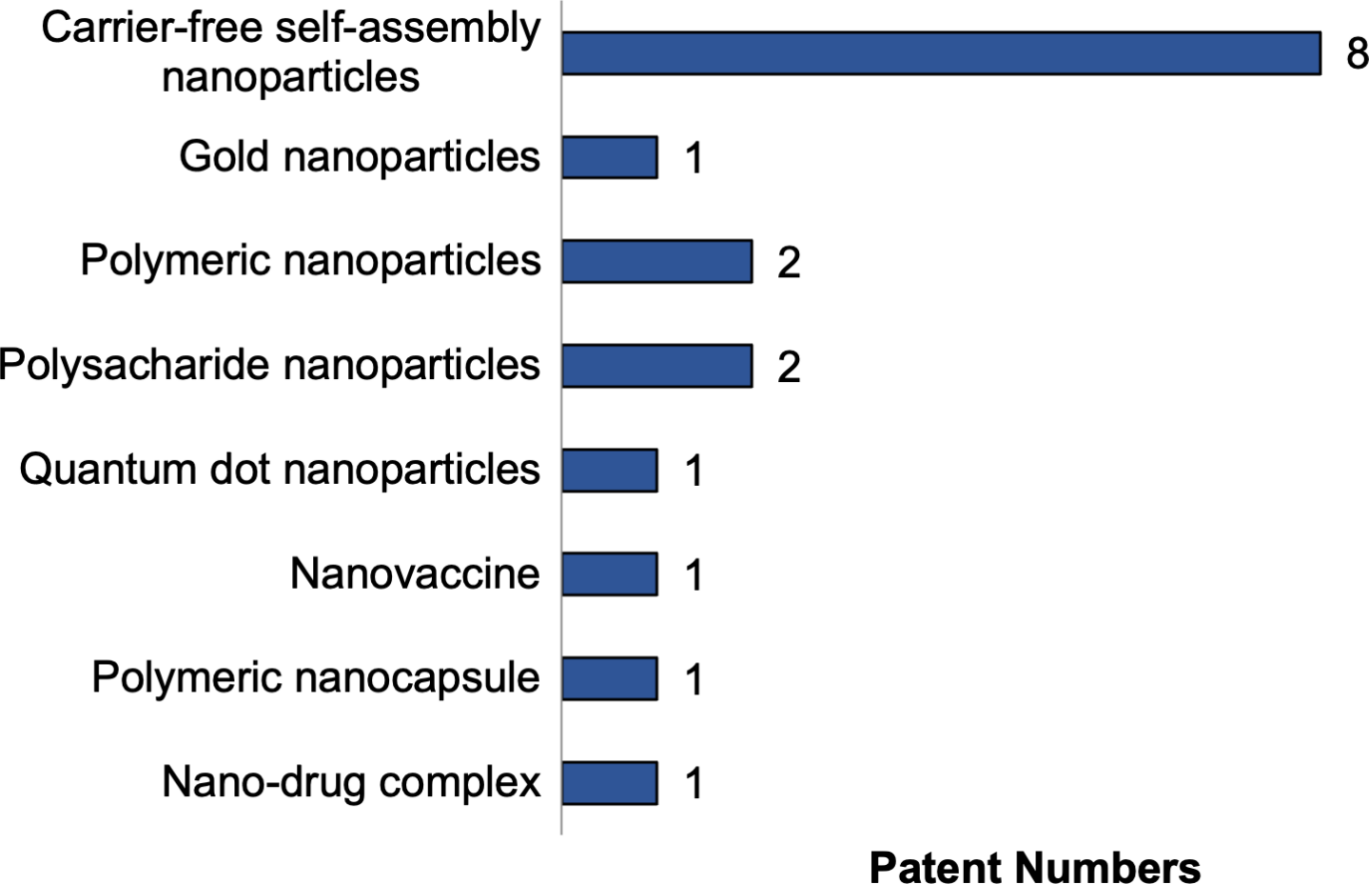
Type of technology present in the selected publications.

Figure 4 shows that the patents analyzed primarily focused on carrier-free self-assembly nanoparticle technology, which was present in eight inventions. Other types of nanotechnology, including nanovaccine, polymeric nanocapsules, and nanodrug complexes, were each identified in a single publication. The 14 inventions based on nanoparticles can be subdivided into five groups according to the type used in the product. Patents WO2016178224 and CN117534780 utilized polysaccharide nanoparticles, while patent KR20220169108 used gold nanoparticles, CN427216811 implemented quantum dot nanoparticles, CN367902299 and CN115887415 employed polymeric nanoparticles, and the remaining inventions used carrier-free self-assembly nanoparticles.

The greater number of inventions based on nanoparticles can be attributed to the longer period over which this carrier system has been studied and utilized. Indeed, the scientific literature contains reports dating back to the early 2000s that describe the application of nanoparticles in cancer treatment [34]. In contrast, other technologies such as nanovaccines and nanocapsules are relatively recent developments, which may account for their lower representation in the current patent landscape [35].

Following this analysis, polysaccharide nanoparticles have high biocompatibility and the ability to encapsulate therapeutic molecules, representing an alternative to the use of natural products [36]. However, although polysaccharides are often studied in pharmaceutical systems, this has not been reflected in patent searches, as only two publications were found. Self-assembled nanoparticles are structures with adapted particle interactions to achieve desired purposes [37]. In the pharmaceutical field, these nanoparticles have potential as vehicles for anticancer drugs due to their biocompatibility, cellular absorption, and slow release of drugs [38]. Polymeric nanoparticles are colloidal polymer systems used as drug carriers for targeted therapies and diagnostics [39]. Gold nanoparticles have properties such as chemical reactivity, anti-inflammatory effects, and protein-binding abilities, while quantum dot nanoparticles are fluorescent semiconductor compounds that can act as drug carriers [40–41].

### Nanotechnology, natural products, cancer, and immunotherapy

Natural products are chemicals produced by living organisms such as microbes, marine organisms, animals, fungi, and plants. They are widely used as therapeutic agents to treat diseases and maintain health and wellness [42]. In recent years, treatments involving natural products have seen significant growth in academia. Historically, natural products have made up 23.5% of approved medicines over the past 40 years. However, current technological development and an interdisciplinary approach have led to the more widespread implementation of these natural products in pharmaceutical research, as they are preferred for their favorable therapeutic efficacy, low adverse effects, and cost-effectiveness compared to synthetic products [43–45].

The use of natural products in cancer treatment and immunotherapy is mainly represented by the use of certain classes of compounds. Among them are saponins, which can remodel the tumor microenvironment, polysaccharides, such as lentinan, which increase immune cell activity, and polyphenols that can modulate immune checkpoints, such as curcumin seen in patents CN111202719 and US240447339, and terpenoids with direct anticancer activity, such as paclitaxel, observed in patent CN111202719 [46–50].

Moreover, natural products play a promising role in cancer treatment and immunotherapy. Their potential effects include tumor cell death, inhibition of proliferation, increased autophagy, and enhanced immune system response [51]. Furthermore, natural products can also promote the regulation of immune cells and cytokines, increasing immunogenic cancer cell death, natural killer cell activity, and dendritic cell activity. Other effects include the immunomodulatory function of natural products, which enhances their benefits as immune checkpoint inhibitors [52].

Based on this, analyzing the bioactive compounds responsible for these effects, such as saponins, polysaccharides, flavonoids, and natural products from traditional Chinese medicine like baicalin and wogonin, which reverse the immunosuppressive environment of tumors [48,50]. In addition to their active effects, natural products can be used as adjuvants and aids to conventional treatments, helping to boost antitumor capacity and reduce adverse effects [53–54].

**Figure 5:**
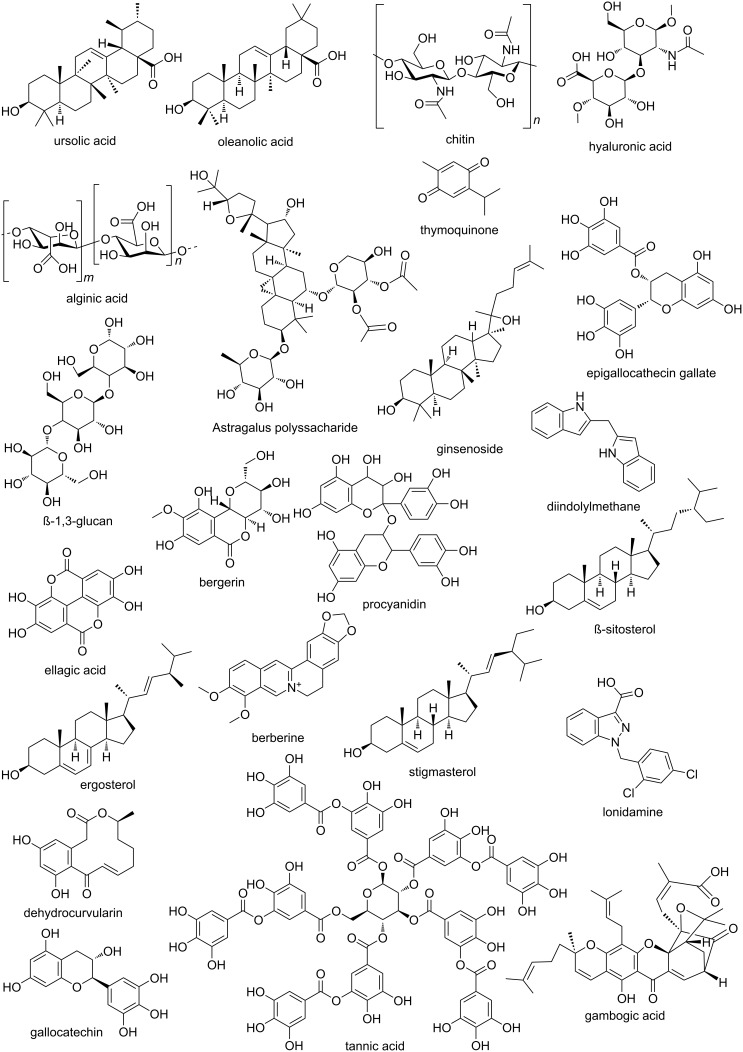
Chemical structures of selected compounds. Source: Made by the authors using ChemDraw Professional 16.0.

Therefore, the use of natural products for cancer treatment and immunotherapy formulation presents a promising option for the pharmaceutical industry. One of the main technologies implemented in pharmaceutical development for incorporating natural products in cancer and immunotherapy is nanotechnology, as it presents physicochemical benefits for drugs. Among the advantages, nanotechnology can eliminate the limitations of using bioactive compounds in cancer treatment formulations, leading to increased bioavailability and pharmacokinetics [55]. The mechanisms behind the improvement in bioavailability and pharmacokinetics are based on increased solubility, observed, for example, by Chittasupho et al., improved permeation through biological barriers, as seen in the work of Huang et al., and protection against premetabolism and early elimination, as analyzed by Miguel et al. [56–58]. Additionally, the controlled release of the drug provided by pharmaceutical technologies can help reduce the toxicity and adverse effects of cancer treatments [59]. Consequently, the implementation of nanotechnology is seen as fundamental to the production of antineoplastic and immunomodulatory drugs from natural products. Table 1 shows the main information extracted from the patents, while Figure 5 shows the chemical structures of the active compounds used [60–76]. Some patents lack information about their preclinical formulation evaluation tests, such as data on the species, strains, and doses used. This reduces the reliability of the results, as it limits access to the reliability, reproducibility, and predictive value of the findings. Especially in the field of oncology, it is essential to have information about the procedures and results, as this allows researchers to analyze whether the study models represent human biology, whether the results are biased, and whether the research can enable the prediction of clinical outcomes [77–79].

**Table 1:** Selected patents that use nanotechnology and natural products for cancer treatment and immunotherapy.

Patent number		Country/year of publication		

nanotechnology/physico- chemical properties^a^	natural product	biological activity	in vitro and in vivo tests	outcomes

WO2016178224 [60]		WO/2016		

polyssacharide nanoparticle PZ: 300 nm ZP: Negative surface charge at physiological pH (7.2–7.5)	hyaluronanic acid and alginate	targeting and uptake by certain tumor cells, peptides, integrin receptors, growth factor receptors and antibodies	in vitro cytotoxicity and antitumor efficacy were assessed using CT26 and MDA-MB-231 cancer cells; in vivo antitumor activity was evaluated in female athymic nude mice with MDA-MB-231 cells	significantly reduced cell viability, reduced tumor size, low cardiotoxic effect and lower IC_50_ values compared to the free drug

US240447339 [61]		US/2017		

polymeric nanocapsule PZ: 100–500 nm PDI: <15% (monodisperse) ZP: +5 to +30 mV or −30 to −5 mV EE: Not specified DL: Not specified RP: Controlled release via shell properties (pore size: 0.01−2 nm, viscosity: 3−6 mPa·s)	diindolylmethane and ellagic acid	anticancer efficacy	in vitro CAM model was used for pancreatic cancer cell implantation; additional in vitro tests were conducted with SUIT-2, colon, breast, ovarian, and bladder cancer cells	Encapsulated forms inhibited cell viability by 50–70%, showing a significantly higher effect than unencapsulated forms. They also reduced tumor size and angiogenesis.

CN222367609 [62]		CN/2017		

carrier-free self-assembly nanoparticle PZ: 115 nm	epicatechin gallate, gallocatechin, epicatechin and procyanidin	epicatechin gallate, gallocatechin and epicatechin: inhibitory activity on telomerase; procyanidin: inhibition effects on cancer cells and induced apoptosis mechanism	in vitro cytotoxicity test, staining of dead living cells, cell migration detection and targeting test with MCF-7 cell strains	better killing effect on breast cancer cells MCF-7 compared with the individual nanoparticles, effectively targeting breast cancer cells effect and inhibition of migration of breast cancer cells MCF-7

CN225561345 [63]		CN/2018		

nanodrug complex N.S.	tannic acid, catechin, epigallocatechin or procyanidin	remotion of free radicals in the body, resistance of oxidation, inflammation and cardiovascular diseases, preventing and treating cancers.	in vitro survival rate test of MBA-MD-231 cells; in vivo test with mice inoculated with MBA-MD-231 tumors and with bone tumors	significant inhibitory effect on cancer cells in vitro, tumor tracing effect, reduction of the tumor size, with inhibition of the growth of tumor cells, inhibitory effect on bone tumors

CN109846857 [64]		CN/2019		

carrier-free self-assembly nanoparticle PZ: 273 nm (β-sitosterol), 154 (ergosterol), 585 nm (stigmasterol) PDI: – ZP: – EE: – DL: 3.4%, 2.4% and 4.3% RP: –	sterol natural products like β-sitosterol, ergosterol or stigmasterol	anticancer, and synergistic effects with photosensitizers	in vivo effect with Balb-c female mice with 4T1 tumor cells; in vitro anticancer activity with murine 4T1 and human MCF-7 breast cancer cells	increased phototoxicity compared to the single drug, and synergistic anticancer effects, with reduction of the tumor volume higher than the drugs alone

CN111202719 [65]		CN/2020		

carrier-free self-assembly nanoparticle PZ: 150–190 nm EE: 94.41 ± 4.28% (UA-PTX NPs), 58.76 ± 2.54% (OA-PTX NPs) DL: 23.12 ± 1.07% (UA-PTX NPs), 12.95 ± 0.51% (OA-PTX NPs) RP: ≈40% (pH 7.3), ≈30% (pH 5.5) (UA-PTX NPs); ≈18% (pH 7.3), ≈10% (pH 5.5) (OA-PTX NPs)	ursolic acid and oleanolic acid	effects in blocking cell cycles and in reducing tissue damage by chemotherapy	in vitro release experiment: measured at 37 °C and pH 7.3 and 5.5; in vitro cell experiments with MCF-7 cells; in vivo antitumor with 4T1 tumor-bearing mice	ursolic acid and paclitaxel achieved a synergistic effect by inhibiting MCF-7 cells growth, with a significantly higher effect than that of the single drug group and the single carrier group

CN114129571 [66]		CN/2022		

carrier-free self-assembly nanoparticle PZ: 80–120 nm	ursolic acid	low toxicity, high efficiency, and multi-link regulated antitumor properties	in vitro flow cytometry with HepG2 cells; in vivo mouse H22 liver cancer transplant tumor model	synergistic anticancer effect superior to any single therapy, with increase of the water solubility and bioavailability of the drug

KR20220169108 [67]		KR/2022		

gold nanoparticle N.S.	ginseng and black cumin extract	black cumin: anticancer and antiproliferative activities	in vitro test with AGS human gastric cancer cells and toxicity test with macrophage RAW264.7 and HaCaT cells	no serious toxic effects and increased inhibitory activity against AGS cells compared to black cumin alone

CN367902299 [68]		CN/2022		

polymeric nanoparticle N.S.	procyanidin	inhibiting TMEM16A ion channels effect, promoting the inhibition of cancer	in vivo test with L795 lung adenocarcinoma tumor model in BALB/c mice	Good drug targeting and significantly reduced the tumor volumes compared to free drugs.

CN114470229 [69]		CN/2022		

carrier-free self-assembly nanoparticle N.S.	ursolic acid	increase of sensitivity of tumor cells to chemotherapy and synergistic effect	in vitro cytotoxicity experiment with HepG2 cells, and in vitro cellular uptake experiment with LO2 cells and HepG2 cells	A synergistic effect with sorafenib inhibited HepG2 cell proliferation and growth more effectively than individual drugs. It also improved targeting capability

CN115252560 [70]		CN/2022		

carrier-free self-assembly nanoparticle PZ: 100–300 nm PDI: 0.05–0.28 RP: Slow and prolonged release at pH 7.4 and pH 6.5	berberine, lonidamine and gambogic acid	inhibition of respiratory chain complex, hexokinase 2, glycolysis, mitochondrial targeting, cytotoxicity and glutamine metabolism	in vitro cytotoxicity and antitumor effect test with breast cancer cells 4T1 and normal human liver cells, hexokinase activity assay, determination of glutamine content, mitochondrial respiratory chain activity assay, and mitochondrial targeting ability	The treatment exhibited low cytotoxicity, high biocompatibility, and selectivity, enhancing anticancer activity. It inhibited glycolysis, reduced glutamine levels, and decreased mitochondrial respiratory chain activity.

CN117064865 [71]		CN/2023		

carrier-free self-assembly nanoparticle PZ: 150 nm	ursolic acid	inhibition of the differentiation of tumor cells, and immune-activating effect, with stimulation of cytokines	in vitro antitumor HepG2 cells and flow cytometry 293T cells experiment; in vivo effect with Balb-c female mice with 4T1 tumor cells; in vitro anticancer activity with Mouse 4T1 and human MCF-7 breast cancer cells	targeting gene knockout effect and killing effect on tumor cells

CN426774477 [72]		CN/2023		

polymeric nanoparticle PZ: 100 nm	astragalus polysaccharide	antiviral, antitumor, anti-aging, anti-radiation, anti-stress, and antioxidant effects	in vitro immune checkpoint inhibitor testing, NF-κB pathway blocking effect and cytotoxicity test with B16F10 tumor cells	promotes delivery of PD-L1 antibodies, great immune checkpoint target effect, toxic and side effects reduced, immune activation effect improved, and synergistic effect achieved

CN115671277 [73]		CN/2023		

nanovaccine PZ: 170–300 nm RP: sustained release with acid sensitivity	astragalus polysaccharide	antiviral, antitumor, anti-aging, anti-radiation, anti-stress, and antioxidant effects	flow cytometry with antibodies; in vitro BMDC maturation effect test; immunotherapeutic in vivo effect on mouse B16F10 melanoma model and mouse Lewis lung cancer model	upregulation effect on the expression of co-stimulatory molecules, lower tumor size compared to the control group, treating tumor effect, activation of immune responses

CN427216811 [74]		CN/2023		

quantum dot nanoparticle PZ: 2.68 ± 0.54 nm PDI: 14.68 ± 0.99 mV EE: 95.8% RP: 18.23% (pH 7.4, laser), 21% (pH 5.0, laser)	berberine	anti-inflammatiory, protection of the liver, improvement of immunity and antitumor activity	in vitro cytotoxicity test with L929 cells and in vitro photothermal antitumor effect on LLC cells	photothermal therapy effect and a chemotherapy effect under laser irradiation, efficiently killing LLC cells, having synergistic effect

CN115887415 [75]		CN/2023		

polymeric nanoparticle PZ: <100 nm PDI: 0.012 ZP: >20mV EE: 53.28% DL: 10.23% RP: Continuous release	dehydro- curvularin	inhibition of the growth, reproduction migration, transformation and cloning of tumor cells	in vitro cytotoxicity and cellular uptake test with MCF-7 cells and MM-231 cells; in vivo tumor inhibition effect in female Balb/c mice with a breast cancer tumor (4T1 cell) model	tumor cell growth inhibition effect better than that of free drugs, good targeting, promotion of release of the drug inside the cells, and slowing effect of the growth of tumor volume

CN117534780 [76]		CN/2024		

nanopolysaccharide nanoparticle PZ: 125–300 nm	chitin and β-1,3-ᴅ-glucan	stimulate immune cells and improve its interaction with cell receptors in the body	in vitro antitumor HepG2 cells experiment, immunoassay with RAW 264.7 macrophages	significantly increased cell inhibition rate and immune-promoting effects on cells by increasing cytokines levels

^a^Abbreviations: DL, drug load; EE, encapsulation efficiency; N.S., not specified; NPs, nanoparticles; OA, oleanolic acid; PDI, poly dispersion index; PTX, paclitaxel; PZ, particle size; RP, release profile; UA, ursolic acid; ZP, zeta potential.

By analyzing the chemical structures of the natural compounds illustrated in Figure 5, it is possible to identify their relationships with cancer control and immunotherapy. For instance, terpenoids such as ursolic acid, oleanollonidamine, exhibit variations in selectivity and potency against cancer cells, depending on the presence of methoxylations at positions 3 and 20 [80–82]. In phenolic compounds such as catechins, procyanidin, tannic acid, and thymoquinone, the arrangement and presence of the aromatic ring, along with hydroxy, methoxy, and carboxyl groups, directly influence the antioxidant activity of these substances and their ability to induce apoptosis [83–85]. Furthermore, in polysaccharides such as chitin, hyaluronic acid, and alginate, factors such as molecular weight, degree of branching, monosaccharide composition, and processes like sulfation or phosphorylation significantly influence their ability to modulate the tumor microenvironment and activate the immune system [86–88]. Finally, alkaloids and their derivatives, such as lonidamine, exhibit variations in selectivity and potency against cancer cells, depending on the presence and position of hydroxy, methoxy, or alkyl groups, as well as modifications to the nitrogen atom [89–91].

Patents WO2016178224 and US240447339 show robust results, with solid data both in vitro and in vivo, suggesting high efficacy compared to the free drug and lower toxicity. Patents CN222367609 and CN225561345, although their results are limited to specific cell lines, also show good results in terms of targeting and tumor inhibition.

Following this perspective, patents CN111202719, CN114470229, and CN115252560 represent advances in tumor therapy, since they combine cell cycle blockade and inhibition of metabolic pathways, which are considered complex cellular mechanisms. Nevertheless, patent CN109846857 stands out for combining the natural compound with photosensitizers, presenting a synergistic effect that is better than the isolated effect of the substances, with a methodology not seen in the others formulations. Finally, patents CN117064865, CN426774477, and CN115671277 combine immunotherapies containing immunomodulators and checkpoint inhibitors to achieve a synergistic effect and consequently expand the clinical potential of nanotechnology containing natural products.

Patent KR20220169108 was discussed in the article published by Dhandapani et al., which addresses the synthesis, physicochemical characterization, and therapeutic evaluation of nanotechnology [92]. The formulation of patent CN114470229 was discussed in an article by Guo et al., which showed that the nanoparticle, as in the patent, achieved better solubility, synergistic effect, and better targeting [93]. In addition, the article published by Li et al. discussed patent CN115671277 [94].

### Nanoparticles

Nanoparticles are materials with at least one dimension ranging from 1 to 999 nm [95]. This technology possesses a high contact surface, a high concentration of surface-active centers, and low toxicity due to promoting a reduction in the dose of its active ingredients [96]. These characteristics have made nanoparticles widely used in medicines, medical applications, and the food industry, as they provide increased shelf life, improved drug delivery, and enhanced therapeutic efficacy [97]. Various methods, such as chemical reduction, microemulsion and inverse microemulsion, hydrothermal method, seeding, sonoelectrodeposition, and coprecipitation can be implemented for the synthesis of nanoparticles [98].

In the context of cancer treatment, nanoparticles promote enhanced biocompatibility, reduced toxicity, and increased stability and permeability, which can help overcome challenges like multidrug resistance [23]. Moreover, this technology enables better encapsulation of active pharmaceutical ingredients (APIs), contributing to improved drug delivery to the tumor region and promoting new therapeutic approaches for cancer treatment [99]. In the field of immunotherapy, nanoparticles can increase antitumor immunity, reduce immune system evasion, suppress tumor growth, and diminish metastasis by ameliorating the tumor microenvironment [100]. Additionally, this technology promotes the activation of cytotoxic T cells, enhances antigen presentation, facilitates immune cell trafficking, and reduces toxic side effects on non-target cells [101–102]. However, nanoparticles have disadvantages, such as lack of routes of administration, concerns about crossing biological barriers, and tempering biodistribution. In addition, they can cause inflammatory and oxidative effects, posing a risk to the lungs, liver, and kidneys [27].

Regarding the use of natural products, nanoparticles can increase the bioavailability, pharmacokinetics, and selectivity of compounds toward cancer cells, thereby improving their solubility and delivery [103]. Moreover, nanoparticles can be subdivided into various structures, such as carrier-free self-assembly nanoparticles, polymeric nanoparticles, polysaccharide nanoparticles, gold nanoparticles, and quantum dot nanoparticles.

### Carrier-free self-assembly nanoparticles

Carrier-free self-assembly nanoparticles are formed spontaneously from the organization of active or natural compounds, without the presence of a carrier material or excipients. Their stability is due to the presence of intermolecular interactions, such as electrostatic forces, hydrogen bonding, π–π stacking, and hydrophobic interactions [104–105]. Among the benefits of this technology are reduced toxicity and immunogenicity, as well as less complexity, since they do not implement synthetic carriers in their composition.

However, carrier-free self-assembly nanoparticles may have some limitations, such as the limitation of natural products used, since the compounds must have properties that contribute to a stable formulation, difficulty in controlling physicochemical characteristics, such as particle size, which can affect reproducibility and scalability, and problems related to rapid clearance and interaction with non-target tissues, mainly due to the absence of a carrier substance [104,106–107]. In short, this technology reduces adverse effects and environmental risks, mainly due to the greater use of natural products over synthetic ones, and promotes increased stability and drug load efficiency [104,108].

For example, patent CN114129571 (2022) describes the formation of metal–organic co-assembly nanoparticles without a carrier. The formulation contains sorafenib, a molecularly targeted anticancer drug, ursolic acid as an API, and iron ions. Ursolic acid, a naturally occurring pentacyclic triterpenoid, exhibits a variety of biological activities and potential health benefits, and it can be found in herbs, fruits, and traditional Chinese medicinal plants [109]. The compound can be extracted through various methods, such as maceration, heat reflux, and Soxhlet and ultrasonic extraction, with dichloromethane considered the most efficient and selective solvent [110]. Ursolic acid exhibits anti-inflammatory, antimicrobial, antiviral, antioxidant, and antiproliferative properties, making it a potential antitumor agent [111]. It inhibits tumor cell proliferation, prevents metastasis and angiogenesis, and induces cell death [112]. The therapeutic mechanisms involve inhibiting cell proliferation by disrupting the lysosomal system, increasing pH, and altering the lipid profile of cancer cells [113]. Additionally, ursolic acid can inhibit the differentiation of Th17 cells from the STAT3/RORβt pathway and the migration of these cells by down-regulating CXCL9/10 expression in Schwann cells [114]. Particle size and stability of the formulation in the patent were characterized using scanning electron microscopy (SEM). The nanoparticles demonstrated enhanced uptake in HepG2 cells, as confirmed by flow cytometry and confocal microscopy, and achieved a tumor inhibition rate of 75.9% in 15 days, significantly higher than ursolic acid and sorafenib alone [66].

Patent CN117064865 (2023) claims a nano bionic CRISPR/Cas9 drug co-assembly system to treat liver cancer. The technology is composed of ursolic acid as an API, a Cas9 ribonucleoprotein (RNP) complex that targets the PD-L1 gene, a cell-penetrating peptide, and a tumor cell membrane derived from HepG2 cells. Characterization of the nanoparticles showed that the nanocomplex was stabilized by hydrogen bonds, van der Waals forces, and hydrophobic forces. In addition, confocal microscopy, gene editing efficiency, MTT assay, and flow cytometry tests were carried out to observe the therapeutic activity of the technology. The results demonstrated that the nanoparticles increased targeting and internalization, had a knockout rate of 80%, and exhibited better inhibition of HepG2 cell proliferation compared to the individual components, revealing a synergistic activity for cancer treatment [71].

Patent CN114470229 (2022) describes carrier-free double-drug self-assembled nanoparticles for treating liver cancer. This technology contains indocyanine green, a cell-penetrating peptide, a nucleic acid aptamer, sorafenib, and ursolic acid as an API at 4 mg·mL^−1^ in methanol. Physicochemical tests showed that the nanoparticles have a spherical shape, confirmed by atomic force microscopy (AFM), and are stable in ultrapure water and Dulbecco’s modified eagle medium (DMEM) with 10% FBS. Procedures to evaluate the nanoparticles’ antitumor activity included fluorescence intensity and combination index to determine drug synergy. The results indicate the nanoparticles exhibit a synergistic effect, effectively target HepG2 cells as observed by fluorescence, and show a higher inhibition of cell proliferation than the free drugs, demonstrating superior therapeutic potential [69,115–117].

Furthermore, nanoparticles containing antineoplastic drugs have demonstrated the ability to act against cancer. They do so by inhibiting the cell cycle, blocking the depolymerization of microtubules, and stimulating the development of reactive oxygen species (ROS) [118]. Hyaluronic acid can inhibit local recurrence and distant tumor growth when used in immunotherapy scenarios in conjuction with CAR-T cells and anti-PDL1-conjugated platelets [119]. Transmission electron microscopy (TEM) and dynamic light scattering (DLS), and UV–visible (UV–vis) spectroscopy were used for characterization. The nanoparticles showed increased cellular uptake compared to free PD-L1, suppression of the NF-κB pathway as indicated by reduced PHO-P65 protein expression, and enhanced tumor inhibition due to immune activation and targeted delivery [72].

Patent CN222367609 (2017) describes targeted amphiphilic nanoparticles composed lecithin, procyanidine, and doxorubicin condensated with epigallocatechin gallate in *N*-hydroxysuccinimide solution, which were developed to inhibit breast cancer. The procyanidine and epigallocatechin gallate act as APIs, while lecithin is an excipient. Catechins and their derivatives are natural polyphenolic compounds that are found in foods such as chocolate, red grapes, wine, and apples, as well as especially in green tea from *Camellia sinensis* (*C. sinensis*) and *C. assumica* [120–121]. These compounds present remarkable properties in treating and preventing cancer, including antioxidant, anti-inflammatory, and antiangiogenic effects, as well as inhibitory effects on protein kinases. This results in cancer cell apoptosis, suppression of proteinases, strong inhibition of telomerase, and inhibition of cancer cell migration, invasion, and metastasis [122–123]. The patent characterizes the NPs through DLS and TEM, confirming a uniform size distribution. Additionally, in vitro studies using MCF-7 breast cancer cells demonstrated inhibition of cell proliferation, with the NP inducing apoptosis through their targeted mechanism [62].

Patent CN115252560 (2022) describes self-assembled nanoparticles composed of berberine, lonidamine, and gambogic acid in a 3:1:4 ratio, all acting as APIs, encapsulated with vitamin E polyethylene glycol succinate (VE-TPGS), an excipient, for stability. *Berberis vulgaris* and *B. aquifolium* are some of the main sources of berberine, a quaternary isoquinoline alkaloid compound with a range of pharmacological properties, including antioxidant, anti-inflammatory, antimicrobial, and, most importantly, antitumor effects [124–125]. The drug’s potential for cancer treatment lies especially in its activation of the apoptotic pathway and the blocking of HIF-1α expression [126].

This combination of drugs in the formulation yields multiple mechanisms against cancer. Berberine provides mitochondrial targeting, lonidamine inhibits hexokinase, and gambogic acid offers cytotoxicity. The nanoparticles showed inhibition of the proliferation of breast cancer cells, with lower cytotoxicity to normal liver cells compared to standard drug and free drug. This is due to the nanoparticles’ enhanced permeability and retention, which improves tumor-specific targeting. Mitochondrial membrane potential investigation using 2 μg·mL^−1^ of NPs for 12 h determined that the formulation induced early apoptosis in the targeted cells [70].

Patent CN111202719 (2020) utilized a nanosystem containing ursolic acid and oleanolic acid, paclitaxel, curcumin, camptothecin, and polyvinyl alcohol as APIs and drug carriers, and methanol, ethanol, and acetone as solvents. Both nanoparticle systems exhibited high biocompatibility and low cytotoxicity to normal cells. Additionally, the results demonstrated the technology had a tumor inhibition greater than 70% in mice with a synergistic antitumor effect, significantly improved blood indicators compared to paclitaxel injection (*p* < 0.01), extended circulation, improved targeting to tumor sites, and reduced toxic side effects [65].

CN109846857 (2019) describes the development of a natural photosensitizer derived from chlorophyllin e6 (Ce6). Self-assembled nanoparticles containing sterol compounds, such as β-sitosterol, ergosterol, or stigmasterol, were employed to enhance the stability and bioavailability of the active ingredient. Ce6 has the ability to selectively accumulate in cancer cells, attributed to the increased metabolic activity and permeable vasculature of these cells compared to healthy ones. Upon absorption of light during photodynamic therapy, Ce6 generates ROS, causing damage to the cell membrane, proteins, and DNA of the cancer cells, ultimately leading to their destruction. Additionally, the ROS produced by Ce6 destroys the vascular layer surrounding the tumor, thereby inhibiting its continued growth and stimulating immune cells to recognize and target the mutated cells [127–128]. Regarding the natural compounds mentioned in the patent, sitosterol is particularly noteworthy. Preclinical studies have demonstrated that sitosterol can induce cell cycle arrest, regulate oxidative stress, enhance metabolic reprogramming, inhibit invasion and metastasis, and modulate immunity and inflammation [129]. The developed nanoparticles underwent physicochemical characterization by SEM, UV–vis spectroscopy, and encapsulation testing, in which the nanoparticles demonstrated stability, spherical shape, and homogeneous size distribution. The use of nanoparticles with sterol compounds associated with Ce6 yielded higher tumor inhibition rates compared to the isolated compounds, especially the nanocarrier with ergosterol, which reached a rate of 86.4% [27,64,130–133].

### Polymeric nanoparticles

Polymeric nanoparticles are solid colloidal systems of synthetic or natural polymers, which can be organized in hollow, occluded, multilobed, and core–shell structures, depending on their thermodynamic and kinetic characteristics [130]. Polymeric nanoparticles can be synthesized using methods such as direct polymerization of monomers, nanoprecipitation, solvent evaporation emulsification, dispersion of preformed polymers, or salting-out [27]. For cancer and immunotherapy, polymeric nanoparticles offer advantages such as biocompatibility, stimulation of T cells, controllable size, and protection of the tumor environment [131]. They also help by reducing adverse effects, increasing antitumor response, as well as increasing solubility and precision in drug delivery [132–133].

Seeking a new pharmaceutical application for procyanidines (PCs) as APIs, the patent CN367902299 (2022) describes photo-thermally responsive PC-loaded polymeric nanoparticles designed to prevent and/or treat lung adenocarcinoma by inhibiting TMEM16A. TMEM16A is a calcium-activated chloride ion channel that appears to be a suitable biomarker and target for lung cancer treatment. PCs, also known as proanthocyanidin, are phenolic compounds of the flavonoid family and are a class of natural polymers formed by catechins and epicatechins [134]. They exhibit a variety of bioactive properties, including anti-inflammatory, antimicrobial, cardioprotective, and neuroprotective effects, which could contribute to cancer prevention and treatment [135]. In cancer therapy, PCs show potential for inhibiting cancer cell proliferation, inducing apoptosis, modulating oxidative stress, suppressing angiogenesis, and interfering with signaling pathways involved in tumor progression [136]. The formulation includes temperature-responsive and non-temperature-responsive amphiphilic molecules (weight ratio of 7:3). UV–vis spectroscopy, TEM, and DLS experiments demonstrated that administration of 14.37 mg·kg^−1^ of the nanoparticles inhibited lung adenocarcinoma proliferation and migration after 24 h [68].

Similarly, patent CN426774477 (2023) developed an immune checkpoint inhibitor nano-delivery system using natural polyphenols. The formulation contained sulfhydrylated hyaluronic acid (HA-SH), which acts as an adjuvant carrier, and epigallocatechin gallate (EGCG) as an API to form HA-EGCG, as well as manganin, FeCl_3_, and the PD-L1 antibody. Hyaluronic acid is a non-sulfated glycosaminoglycan component of the extracellular matrix and has diverse biomedical applications [115]. It can be obtained through the fermentation of bacteria and yeasts, as well as animal sources, using chemical, enzymatic, and combined extraction methods [116–117].

CN115887415 (2023) describes a methoxypoly(ethyleneglycol)-poly(lactic-*co*-glycolic acid) (mPEG-PLGA) nanocarrier containing dehydrocurvularin; the latter is the API of the formulation and exhibits sustained release. Dehydrocurvularin is a natural benzenediol produced by many fungi as a secondary metabolite. Its therapeutic activity is focused on antitumor capacity through its synergistic activity with drugs, increasing efficacy and tumor targeting capacity [127,137]. The formulation combines mPEG-PLGA, acetonitrile, and Tween-80. The nanoparticles were obtained by precipitation and freeze-drying and were analyzed by HPLC and DLS. Additionally, the in vivo studies performed in a mice model with breast cancer tumor (4T1 cell) showed that the drug exhibits a level of biosafety while reaching 44% tumor inhibition [75].

### Polysaccharide nanoparticles

Polysaccharides are a class of polar polymers frequently employed in polymeric systems and nanotechnologies, including the creation of polysaccharide nanoparticles [28]. Substances such as chitosan, hyaluronic acid, alginate, starch, and their derivatives are most commonly used in nanoparticles for therapeutic applications [138]. Organic solvents, ionic liquids, inorganic strong alkalis and acids, enzymes, and hydrothermal treatment are used to obtain polysaccharide nanoparticles [139]. Consequently, polysaccharide-containing nanoparticles offer an alternative for cancer treatment, promoting antitumor immune responses with reduced toxicity and fewer side effects [140]. Moreover, this technology can be applied to various cancer therapies, such as chemotherapy, photothermal therapy, photodynamic therapy, gene therapy, and immunotherapy [141].

For instance, patent WO2016178224 (2016) describes the development of anionic polysaccharide nanoparticles designed for the delivery of anionic small-molecule anticancer drugs. The formulation consists of anionic polymers, such as hyaluronic acid (HA), alginate (Alg), HA-sulfate, and Alg-sulfate, which acts as adjuvants for drug delivery, as well as anionic small-molecule drugs, including methotrexate (MTX) and doxorubicin (DOX), and divalent cations like calcium (Ca^2+^). Alginate is a cross-linked polymeric network derived from algae and shows potential for cancer treatment due to its improved bioavailability, sustained release, and environmentally benign properties [142–143]. The tests showed that the nanoparticles containing MTX and DOX were around 400 times more effective and yielded higher cytotoxicity in CT26, MDA-MB-231, and NAR cancer cells, compared to the free drugs [60].

CN117534780 (2024) uses chitosan–glucan nanopolysaccharide complexes (CGCs) obtained from fungi to prepare antitumor and immunomodulatory drugs, with chitosan–glucan being an API. Chitosan–glucan is a biopolymer complex composed of chitosan, a deacetylated derivative of chitin, and glucan, a polysaccharide commonly found in fungal cell walls, cereals, and seaweed. Chitosan possesses a combination of properties that include antioxidant and antitumor effect, giving it potential to prevent and/or treat cancer by stimulating apoptosis [144]. Meanwhile, glucan presents potential in cancer therapy due to its immunomodulatory influence, which can increase the recruitment of neutrophils or infiltration of CD4^+^ T cells to destroy tumors [145]. The process of nanosizing the CGCs in the patent involves shearing at high speed and ultrasonic cycles followed by freeze-drying. This resulted in a yield of 12.73% and a purity of 96.32%. The increased surface area due to the reduction in particle size, along with improved interaction with cellular receptors, enables a significantly increase of the cell inhibitory effect (tumor cells were HepG2 and MCF-7), with the complex exhibiting an increased amount of cytokine secretion when used at a concentration of 10 mg·mL^−1^ [76].

### Gold nanoparticles

Gold nanoparticles are nanometer-scale structures composed of a gold core with surface ligands, which can be structured into nanospheres, nanocages, nanorods, and nanoshells [146–147]. There are various manufacturing processes such as vacuum sputtering, biosynthesis, methods based on ultraviolet light, synthesis in reverse micelles, and condensation processes [148]. Gold nanoparticles have great benefits for cancer and immunotherapy, providing increased efficiency and effectiveness by acting as immune regulators, enhancing the delivery of antitumor drugs, and improving biocompatibility, durability, and innate immune responses [149–150]. In addition, gold nanoparticles lead to more selective oncological treatments with biocompatibility and low toxicity and their use in association with natural products is promising [151–152].

The patent KR20220169108 (2022) claims that gold nanoparticles were developed using membrane vesicles from a *Curtobacterium proimmune* K3 strain, isolated from ginseng, and black cumin seed extract, which are presented in the formulation in the form of stabilizing adjuvants. The use of ginseng is based on the fact that its components may have anticancer effects, especially on apoptosis, cell cycle regulation and the PI3K/AKT and MAPK pathways, while black cumin extract is used because its main components, such as thymoquinone, show anti-inflammatory, antioxidant, and immune system stimulating potential [89–90]. The compound was characterized using UV–vis spectroscopy, TEM, DLS, X-ray diffraction (XRD), and Fourier-transform infrared (FTIR) spectroscopy, revealing a polygonal or oval morphology. To evaluate the antitumor effects, cytotoxicity assays, cellular uptake assays, apoptosis detection, ROS production, qRT-PCR, and Western blotting for gene and protein expression, and autophagy inhibition studies were applied. The results showed that gold nanoparticles promoted an increase in apoptosis markers, such as stimulation of p53, Bax, cytochrome c, caspase-9, and caspase-3, as well as a decrease in Bcl2, a greater production of ROS in CC-AuNP-treated cells compared to black cumin alone, and synergistic anticancer effects when combined with rapamycin [67]. Therefore, nanoparticles containing natural products are a promising technology for cancer and immunotherapy with synergistic effects and reduced side effects.

### Quantum dot nanoparticles

Quantum dot nanoparticles are semiconductor structures smaller than typical nanoparticles, ranging from 2 to 10 nm in size. They are composed of heavy metal or inorganic material and exhibit fluorescent activity, making them commonly used for pharmaceutical applications [153–154]. The technology’s composition is characterized by two free functional groups responsible for drug binding, while a semiconductor shell helps reduce toxicity [106]. Quantum dot nanoparticle synthesis processes involve colloidal and plasma systems, which allow for the utilization of bioactive compounds such as fructose, chitosan, citric acid, lignin, cellulose, and starch [155–156]. Furthermore, quantum nanoparticles offer advantages for cancer treatment. They can manipulate emission properties near the infrared region, improving drug targeting and solubility, enhancing tumor detection, and reducing adverse effects on adjacent healthy tissues [157–158]. Regarding the use of natural products, carbon quantum dots, the main type of nanoparticles derived from bioactive compounds, show promising activity for cancer treatment due to their biocompatibility, photostability, and fluorescent characteristics [159].

Bergenin (BER) is a natural compound extracted from cinnabar root. It has been combined with carbon quantum dots to treat lung cancer in the patent CN427216811 (2023). BER is a glycosidic derivative of hydroxybenzoic acid found in various plant families and species worldwide, with *Bergenia purpurascens* and *Ardisia japonica* being its primarily natural sources [160]. The compound and its derivatives have shown antimalarial, trypanocidal, antibacterial, antileishmanial, anti-inflammatory, antioxidant, analgesic, and anticancer activities [161]. BER acts against cancer by inhibiting cell proliferation, inducing apoptosis, suppressing glycolysis, reducing angiogenesis, and promoting the degradation of oncogenic proteins [162–163]. The invention proposes the use of carbon dots (CDs) loaded with BER as an API, with a sustained drug release that increases upon contact with the acidic conditions surrounding tumors. Both non-loaded CDs and free BER exhibited antitumor effects on Lewis lung carcinoma (LLC) cells, which were further improved with CDs–BER. This combination was able to enhance the inhibitory effect on the cells subjected to photothermal therapy, reducing the cell survival rate to 34.5%, while being biocompatible with a survival rate of 80% for normal L929 cells [64–65,74–75,127–129,137,164–175].

### Polymeric nanocapsules

Polymeric nanocapsules (PNCs) are vesicular systems between 1 and 999 nm in size, composed of an internal oily reservoir surrounded by polymeric membranes, non-ionic surfactants, macromolecules, and phospholipids [176–177]. The properties of the pharmaceutical form are governed by size, shape, core structure, and ligands, which can alter factors such as solubility, charge density, hydrophobicity, stability, and binding affinity [178]. The methods for developing PNCs involve appropriate materials and various production techniques, including emulsion polymerization, layer-by-layer self-assembly, interface polymerization, nanoprecipitation, spray-drying, and supercritical fluids [179–180]. This technology offers the ability to encapsulate hydrophilic or lipophilic pharmaceutical drugs, as well as surface modification, making PNCs advantageous for controlled drug delivery systems [132]. PNCs hold potential applications for cancer treatment and immunotherapy. They can provide sustained drug release while decreasing cytotoxicity and modifying tumor retention for tumor treatment. They can also serve as formulations that encapsulate synergistic combinations of drugs and other substances, such as natural products [181–182]. In the field of immunotherapy, PNCs good biocompatibility and the ability to present antigens and activate the T-cell response, while being efficiently distributed in the lymphatic vessels, contributing to the local and systemic antitumor effects of small molecules [183].

However, PNCs face some challenges for therapeutic use, especially regarding the lack of methods for characterizing the shell, which is important for drug release, toxicity due to the use of organic solvents, the tendency to aggregate in aqueous media, and difficulties in sterilizing the formulation [184]. Furthermore, the technology has advantages when associated with natural products, as phytochemicals show antioxidant and anti-inflammatory activity against cancer. For polyphenols, the use of PNCs enhances pro-apoptotic activity, especially against breast, lung, prostate, cervical, and colorectal tumors [185–186]. Therefore, PNCs are a powerful alternative for the use of natural products in cancer and immunotherapy due to their properties such as controlled drug release.

The patent US240447339 (2017) involves the creation of PNCs. These PNCs utilized a range of bioactive compounds, such as diindolylmethane (DIM) and ellagic acid (EA), both as APIs, curcumin, green tea polyphenols, resveratrol, sulforaphane, and tocopherols. DIM is one of the main metabolites of indole-3-carbinol, found in cruciferous vegetables like broccoli, cauliflower, and cabbage; it exhibits antineoplastic properties such as suppression of cell proliferation, migration, and growth [187–188]. Additionally, EA is a phenolic bioactive compound found in fruits, nuts, and herbs, which can help cancer treatment by targeting mitochondrial metabolism and inducing apoptosis through the inhibition of cyclin-dependent kinase 6 [189–191]. Following the development of the patent, the inventors also incorporated biocompatible polymers, such as PLGA and polyethylene glycol, optionally combined with chitosan or polyvinyl alcohol, and an anticancer agent such as cisplatin, doxorubicin, or temozolomide. The characterization of this technology involved analyses of size distribution, surface charge, morphology by TEM, and monodispersity. The data showed that the PNCs promoted a reduction in pancreatic cancer cell proliferation of 50% and in colon cell proliferation of 50–60%, anti-angiogenic effects with suppression of the vascular pattern. Also, a greater suppression with encapsulated drugs compared to free drugs, sustained delivery profiles, improved bioavailability, and reduced rapid clearance were observed [61].

### Nanovaccines

Nanovaccines (NVs) are composed of nanoparticles whose structure contains substances responsible for stimulating the host’s immune system [131]. NVs can be lipid or non-lipid formulations. Although they vary according to the personalization for each patient, they mainly feature micrometer-sized tumor cells and diverse antigens inside and on their surface to promote the stimulation of the immune system [192–193]. Mechanisms such as covalent conjugation of antigen peptides and flash nanocomplexation can be implemented [194–195]. The use of NVs offers advantages like improved target delivery, antigen presentation, strong T cell response, and safety to combat infectious diseases and cancers [196]. Additionally, the NVs can induce tumor cell death, relieve immune suppression, increase antitumor immune activity, and inhibit metastasis [197]. Nevertheless, NVs have technical and biological limitations. Examples include tumor heterogeneity, which reduces the effectiveness of immune responses, problems with antigen delivery, rapid degradation of molecules, tumor evasion processes, and low persistence in the blood. In addition, NVs face challenges related to treatment complexity, high cost, and complications regarding effective cytotoxicity, requiring a combination of different therapeutic approaches [198].

Patent CN115671277 (2023) describes a nanovaccine with the aim of offering an oncological treatment using astragalus polysaccharides as an adjuvant immune response stimulator and drug release promoter. This natural product has potential for anticancer activity, as it can be applied as an immune adjuvant, responsible for inhibiting tumor growth and increasing immune function [199]. The NV was composed of astragalus polysaccharides as delivery vehicles, ovalbumin as a tumor antigen, and microfluidics, which allows for the formation of nanoparticles, size control, and morphology. SEM, TEM, and in vitro release tests were implemented. In order to evaluate the immunomodulatory activity, the researchers carried out migration tests to lymph nodes, activation of dendritic cells, antigen uptake capacity, and antitumor efficacy in C57 mice and nude mice inoculated with B16F10 melanoma and Lewis lung carcinoma model. The outcomes showed that the NV inhibited tumor growth better than vaccines with conventional adjuvants, such as aluminum, and increased the expression of CD80/CD86 co-stimulators in C57 mice [73]. In addition to reducing tumor progression in the Lewis lung carcinoma model, the vaccine had no effect in nude mice lacking adaptative immunity, confirming that its efficacy depends on specific activation of the immune system. Therefore, it can be inferred that the NV containing astragalus polysaccharide showed better efficacy than conventional adjuvants [54].

### Nanodrug complexes

Nanodrug complexes are nanometer-scale assemblies composed of two or more biomolecules, such as active compounds, antibodies, polymers, and polysaccharides, which exhibit unique biological properties [200]. Polysaccharides and proteins are particularly promising vehicles for delivering active compounds as they can protect these molecules and enhance encapsulation, delivery, and release [201]. In the context of cancer therapy, nanocomplexes containing molecules like anti-HER2 antibody, tamoxifen, cisplatin, polyphenols, and DNA have demonstrated increased cellular uptake, improved anticancer efficiency, enhanced tumor penetration, reduced tumor hypoxia, and tumor suppression, while minimizing adverse effects [202–204]. Nonetheless, nanocomplexes face disadvantages regarding their implementation, as they may present low stability, cause accumulation in tissues, and difficulties in controlling drug release [205].

CN225561345 (2018) combines natural polyphenols that act as APIs (tannic acid, catechin, epigallocatechin, or procyanidine), bortezomib, and iron ions to form a traceable boric acid nanodrug complex for tumor tracing and treatment. Tannic acid, a polyphenolic compound consisting of gallic acid esterified to a glucose core, can be found in various plant sources such as grapes, Sicilian sumac leaves, tea, nuts, and oak bark [206]. Tannic acid demonstrates diverse bioactive properties, including antimicrobial, anti-inflammatory, and antioxidant effects, which could play an important role in cancer prevention and therapy [207]. When tested for its pharmacological use in cancer therapy, this compound demonstrated antitumor and molecular targeting capabilities with the capacity to inhibit cell proliferation, induce apoptosis, modulate oxidative stress, suppress angiogenesis, and interfere with signaling pathways associated with tumor growth [208]. The iron ions stabilize the formulation and improve the tracing effect.

The nanodrug complex responds differently to human breast cancer cell lines (MBA-MD-231) based on the pH value, exhibiting stable release in the tumor microenvironment, which has a pH close to 6.5 (ensuring site-specific activation) and a significant inhibitory effect of up to 26.7%. When tested against mouse bone tumors, the BTZ/Ta Iron complex showed an obvious inhibitory effect without causing mice death, which can occur using high concentrations of bortezomib, and avoided damage caused by tumors on healthy bone tissue [63].

### Obstacles to using nanotechnology containing natural products in cancer treatment

Between the idea of a new treatment and its commercialization, there are many obstacles to overcome. When it comes to complex diseases like cancer and technologies such as nanomedicine, there are even more challenges. Cancer is not a simple condition, it is a multifaceted combination involving genetic, molecular, and clinical profiles that vary for each patient. This instance alone can compromise the search for patients to participate in clinical trials. Patient recruitment for an ideal trial with significant results would require a large-scale random selection of people with identical disease parameters, necessitating an adapted clinical trial [209]. This, along with ethical considerations, makes clinical trials a tough process [210].

The commercialization of a medicine also requires scale-up of production to an industrial level and sufficient stability for transportation and storage, which are not easily achieved with all nanotechnologies. This emerging field of therapeutic products still misses robust and well-developed regulatory guidelines from policy-making agencies [211]. There are no standardized guidelines to follow or minimum parameters to meet, as these types of drugs have unique properties and are not equivalent to other categories already consolidated in the market. Bioaccumulation of nanomaterials is a concerning aspect of this category of medicine to be considered. The regulatory guidelines provided by the European Medicines Agency, whatsoever, lack to mention clear reference figures for such accumulation in terms of risk for the environment and leave that assessment of risk-benefit ratio up to the inventors [212]. Moreover, traditional methods of environmental risk assessment may not be sensitive enough to detect the specific impacts of nanomaterials, making this an urgent issue to be addressed in the process of developing nanotechnology medicine [213]. The absence of these guidelines means a lack of criteria for quality control, safety, and efficacy [214]. Therefore, the field of natural products within nanotechnology for cancer treatment is still poorly explored, and those who have developed a product in this area face high costs and uncertainties in regulatory approvals.

Despite US$24.5 billion being invested in research about cancer throughout the years of 2016–2020, its treatment remains at a high cost in development, production, and acquisition, which compromises patient care [215–216]. High expenses are associated with not only the complexity of cancer, but also of nanotechnology and natural product acquisition. Isolation and assuring purity of natural compounds demand a variety of solvents and specialized resources, which results in an increase in value of the final product but also production of waste. Extraction and purification processes involve multiple steps, such as liquid–solid and partition chromatography and high-performance liquid chromatography (HPLC), each demanding specific solvents and materials to achieve the desired selectivity and purity of the compound [217–218]. Traditional nanoparticle synthesis methods often rely on dangerous chemicals, leading to the release of toxic byproducts and environmental pollution [219]. Additionally, these processes typically require substantial energy input for extraction, purification, and nanoparticle formation, further increasing their environmental footprint [220]. Advanced techniques such as ultrasonication, spray drying, and freeze-drying are energy-intensive processes frequently used to create nanoformulations and are not easily substituted as they determine stability and particle size [221].

Abraxane® is an albumin bonded with nanoparticles of paclitaxel, an alkaloid extracted and isolated from *Taxus brevifolia* bark, that is, a drug based on nanotechnology with a natural product indicated for breast cancer treatment [222–223]. One of the main challenges in using natural products in cancer nanomedicine is poor water solubility, which limits bioavailability and therapeutic efficacy [103]. Traditional paclitaxel formulations required organic solvents to solubilize the compound, leading to significant toxicity and hypersensitivity reactions [224]. Abraxane overcomes this by binding the nanoparticles with albumin, therefore changing the delivery system of the drug rather than the structure of it. Additionally, it indicates a success story in the application of nanotechnology of natural based products for cancer therapy.

## Conclusion

In recent years, the demand for innovations that offer improved safety, fewer adverse effects, and greater efficacy in cancer treatments and immunotherapy has increased. In this way, the use of natural products has shown itself to be growing, associated with the implementation of nanotechnologies that seek to enhance the physicochemical properties of formulations. This review analyzed 17 patents for antineoplastic drugs where nanoparticles were the main technology utilized. The formulations developed were observed to promote tumor cell cycle blockage, inhibit metastasis and tumor growth, stimulate the immune system, and exhibit a synergistic effect with anticancer compounds. These formulations also demonstrated enhanced solubility, stability, and controlled drug release while reducing treatment risks. In conclusion, the combination of natural products and nanotechnology has great potential and offers substantial benefits for cancer treatment and immunotherapy. However, issues like high cost, environmental impacts, complex clinical trials, and unclear or inexistent regulations are obstacles for the successful development and commercialization of these therapies.

## Data Availability

Data sharing is not applicable as no new data was generated or analyzed in this study.
